# Prioritized experience replay based on dynamics priority

**DOI:** 10.1038/s41598-024-56673-3

**Published:** 2024-03-12

**Authors:** Hu Li, Xuezhong Qian, Wei Song

**Affiliations:** School of Artificial Intelligence and Computer Science, Jiangnan University, Wuxi, 214122 China

**Keywords:** Reinforcement learning, Experience replay, Soft actor-critic, Temporal-difference error, Engineering, Mathematics and computing

## Abstract

Experience replay has been instrumental in achieving significant advancements in reinforcement learning by increasing the utilization of data. To further improve the sampling efficiency, prioritized experience replay (PER) was proposed. This algorithm prioritizes experiences based on the temporal difference error (TD error), enabling the agent to learn from more valuable experiences stored in the experience pool. While various prioritized algorithms have been proposed, they ignored the dynamic changes of experience value during the training process, merely combining different priority criteria in a fixed or linear manner. In this paper, we present a novel prioritized experience replay algorithm called PERDP, which employs a dynamic priority adjustment framework. PERDP adaptively adjusts the weights of each criterion based on average priority level of the experience pool and evaluates experiences’ value according to current network. We apply this algorithm to the SAC model and conduct experiments in the OpenAI Gym experimental environment. The experiment results demonstrate that the PERDP exhibits superior convergence speed when compared to the PER.

## Introduction

Reinforcement learning is an approach to sequential decision making aimed at optimizing an agent’s learning strategy during its interactions with the environment to achieve maximum expected cumulative reward^1^. In recent years, reinforcement learning has gained significant popularity across various domains. Different scenarios require the careful design of state spaces, action spaces, and rewards to effectively simulate the sequential decision making process inherent in reinforcement learning. Despite the remarkable successes achieved by reinforcement learning, challenges such as low data utilization efficiency and limited generalizability persist^2^.

There is a notable issue with the continuous experimental samples get from the environment, as they tend to exhibit strong correlations, which contradicts the requirement of deep neural networks for independent and identically distributed data. To address this problem, Lin proposed the concept of experience replay, which has proven to be a reasonable solution^3^^4^.The experience replay mechanism stores experiences in an experience pool and reuses them. This approach effectively mitigates the issues of correlated distribution among samples, while also avoiding sample wastage. However, the sampling strategy of this method is uniform sampling, meaning that it assigns the same probability to each sample without considering their relative importance. In practice, agents often benefit more from learning important experiences.

Since then, many prioritized sampling strategies have been proposed. One of the most well-known strategies is the prioritized experience replay (PER) introduced by Schaul^5^, which improved by experience replay. Schaul^5^ used the time difference error (TD error) as a priority criterion to determine the importance of experiences. Subsequent experimental results combining various models have shown that PER significantly improves the sampling efficiency compared to traditional experience replay^6^. Subsequently, more modifications and improvements have been made to the PER algorithm. Ramicic et al, for instance, employed state entropy as a prioritization criterion, reasoning that experiences containing more unknown information have greater learning potential^7^. Li et alproposed three value measures for experiences and prioritied them, with experimental results validating that the TD error serves as an upper bound for these three metrics^8^. Shivakanth et al proposed a sampling strategy based on the learnability of samples, effectively avoiding repetition of training experience and reducing noise to improve learning efficiency^9^.

All of the above-mentioned algorithms have only single priority criterion, which can lead to some limitations and drawbacks. Firstly, it is highly sensitive to environmental changes, which performing unstable in different environments^10^. Secondly, evaluating all experiences accurately becomes challenging due to the large capacity of the experience pool^11^. In response to these challenges, Xi et al proposed a strategy called High Value Prioritized Experience Replay (HVPER), which combines the state value function and TD error as priority criteria. HVPER mitigates the negative impact of experience with high TD errors near the edge of state space, thereby enhancing learning efficiency and accelerating algorithm convergence^12^. Gao et al considers the immediate reward of experiences and incorporates it as a criterion by linearly combining it with the TD error^13^. To prevent valuable old experiences in the experience pool from being under-sampled for an extended period, Liu et al introduced a dynamic experience replay strategy known as PERMAB^14^. This method aims to dynamically evaluate the contribution of each priority during the training process to prevent the model from achieving a suboptimal performance. However, PERMAB still calculates priority scores in a linear manner and only applies non-linear weighting during the calculation of priority weights.

In this paper, we present a novel approach that improves the efficiency for experience replay. Our method incorporates dynamic adjustment of priority criterion weights during the training process, taking into account recent feedback information. The agent estimates average priority level of the experience pool based on the interaction between sampled experiences and current network. During next training iteration, the sampled experience priority weight are re-evaluated according to the average priority level. We dynamically consider the significance of different criteria and update the priorities based on the adjusted weights.

## Methods

### Preliminary knowledge

#### Knowledge

In online reinforcement learning, updating the network after each interaction with the environment can result in catastrophic forgetting of past experiences. Additionally, the correlation between successive exploration data also brings a challenge to network update. To address these issues, Lin introduced an experience replay mechanism, which storing observed experiences in an experience pool and sampling batches from it during training. This approach has been widely successful when combined with various reinforcement learning algorithms^15^.

Lin’s experience replay method^2^ employs a uniform sampling strategy that treats all experiences equally, which can be considered unreasonable. To address this limitation, Schaul^5^ introduced the Prioritized Experience Replay (PER) algorithm, which assigns individual priorities to each experience stored in the experience buffer. In PER, Schaul utilizes TD error to quantify the amount of information contained in an experience and promotes prioritized training for experiences with higher unknown information. The TD error is defined by the following equation:

1 $$\begin{aligned} \delta = R(s,a, s') + \gamma \cdot v(s') - v(s) \end{aligned}$$

where R(s,a, s’) represents the immediate rewards, s and s’ denote the current state and next states, v(s) is the estimated state value function for state s, and $\gamma$ is the discount factor.

To ensure that every experience is sampled, PER implements a stochastic prioritization approach rather than a greedy strategy. This is important as a greedy strategy might neglect experiences with low TD error, causing them to be undersampled or even forgotten. The sampling probability $P_i$ of an experience is calculated using the following equation:

2 $$\begin{aligned} P_i = \frac{{p_i}^\alpha }{{\sum {p_k}^\alpha }} \end{aligned}$$

where, $p_i = \left| \delta \right| + \epsilon$ represents the priority of experience i. $\alpha$ is used to adjust the degree of priority and $\varepsilon$ is used to prevent the probability from being zero.

This priority-based sampling method may changes the probability distribution of experiences, introducing a bias in estimating the action value function Q(s, a). To address this bias, PER uses importance sampling and the importance-sampling weight $\omega _i$ for experience i is demonstrated by the following equation:

3 $$\begin{aligned} \omega _i = \left( \frac{1}{N} \cdot \frac{1}{P_i} \right) ^\beta \end{aligned}$$

where, $\beta$ is another hyperparameter that controls the degree of correcting bias.

**Figure 1:**
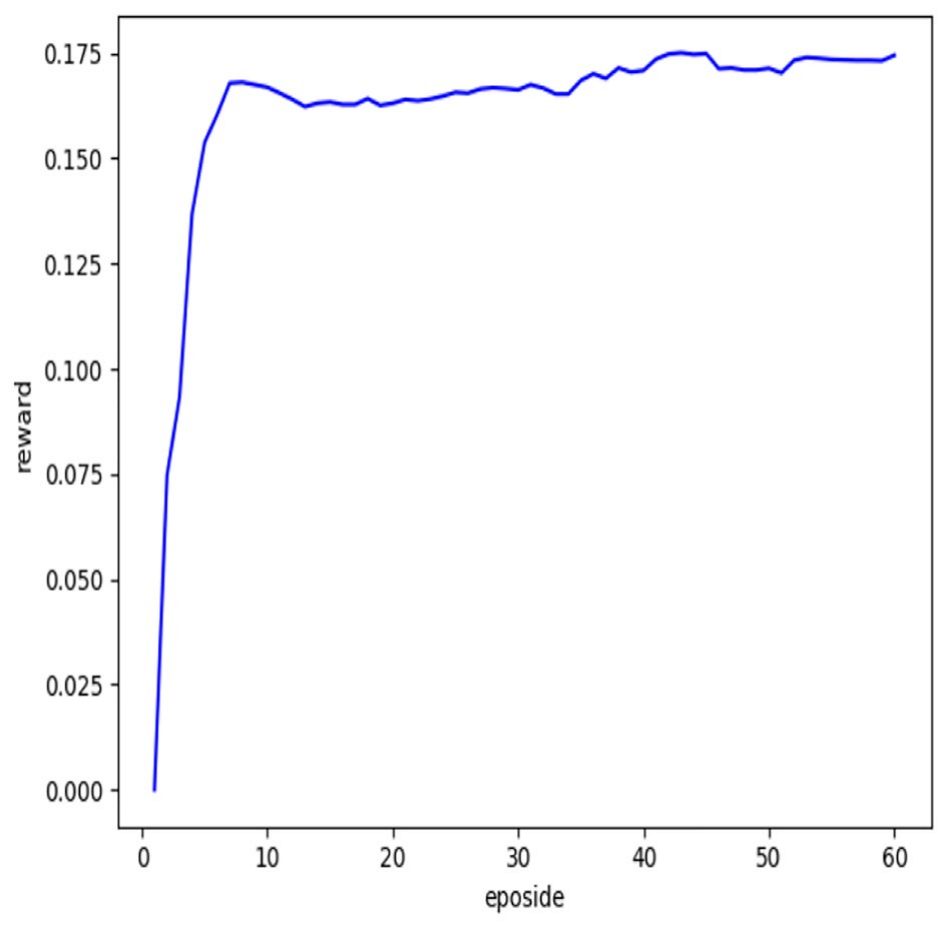
The reward over samples batch with eposidos.

**Figure 2:**
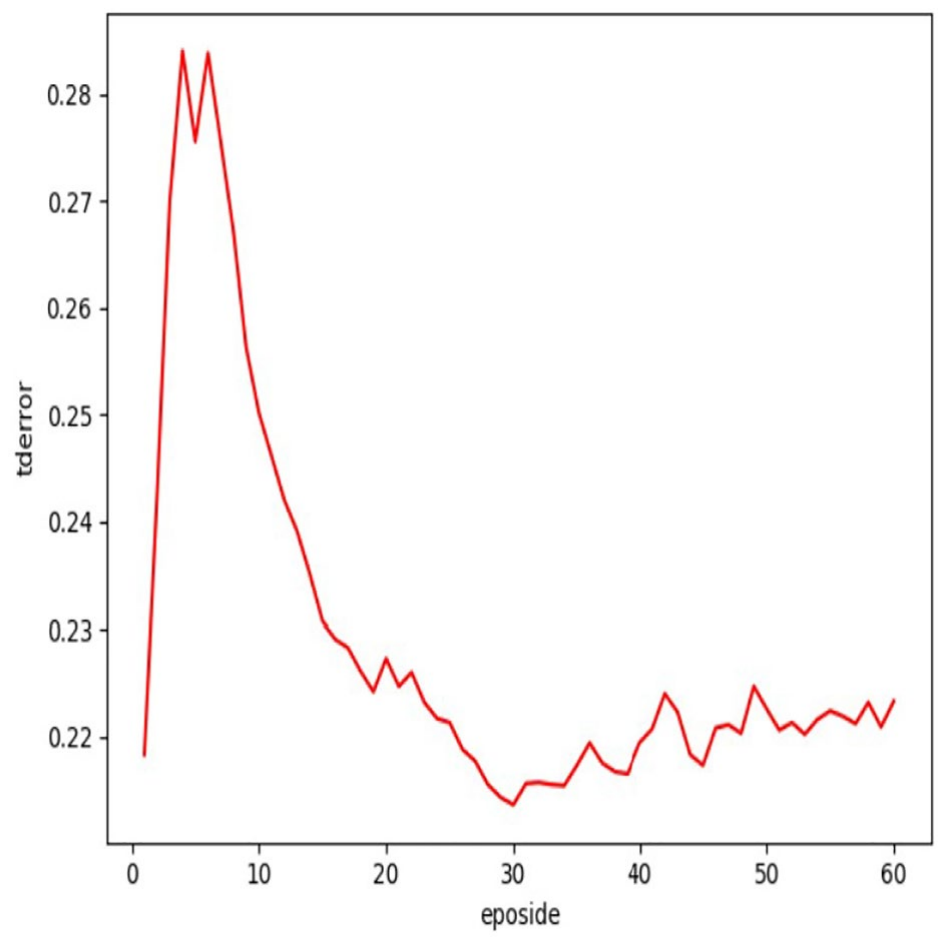
The td-error over samples batch with eposidos.

#### Problem statement

The priority of samples in the experience pool is dynamically changing. With the implementation of stochastic prioritization strategy, all batches will eventually converge, as demonstrated in the Figs. 1 and 2. Nevertheless, different criteria will converge at different stages of training process. It can be observed that the TD-error tends to stabilize after 30 episodes, while the reward converges much earlier.

Efficient sampling experiences from the experience replay buffer is a key challenge in experience replay. To address this issue, we propose an experience replay algorithm evaluating comprehensively the value of experience. By doing so, the agent can sample more valuable experiences from the experience replay buffer, thereby accelerating the learning speed.

### Improved method

In this section, we introduce the PERDP algorithm, which assesses the value of experiences by considering two prioritization criteria: TD-error and reward. This algorithm adjusts the weights of each criterion with respect to the current network.

PERDP uses TD error as a priority because it reflects the uncertainty of an experience. Experiences with higher TD errors indicate a larger discrepancy between the predicted Q-values by the current network, indicating their higher value. The calculation of TD error priority in PERDP is as follows:

4 $$\begin{aligned} p_t = |R_t + \gamma \cdot \max _{a'} Q(S_t, a') - Q(S_{t-1}, A_{t-1})| \end{aligned}$$

where, $R_t$ represents the immediate reward at time step t, $\gamma$ is the discount factor, Q($S_t$, a’) denotes the Q-value for state-action pair ($S_t$, a’), and Q($S_{t-1}$, $A_{t-1}$) represents the Q-value predicted for the previous state-action pair ($S_{t-1}$, $A_{t-1}$).

Despite the advantages of PER algorithm over uniform sampling, it still suffers from some drawbacks. One limitation is that the priorities of experiences in the replay buffer often become outdated because updating the priority of every experience is impractical^16,17^. Additionally, continually sampling high priority experiences can lead to overfitting^18^. Relying solely on a single perspective when evaluating experiences may restrict the model to local optimum^19^. Furthermore, high TD-error experiences are often appear near the edge of state space, as the agent rarely explore in these areas. However, this does not necessarily mean that these experiences can provide significant useful information.

To address this issue, we use another widely used priority criterion called immediate reward $p_r$, mitigating bias through multiple criteria. Immediate Rewards are the most direct reflection of the interaction between agent and environment, especially in sparse reward environments. By considering $p_r$, the agent can account for the historical value of an experience. The calculation of $p_r$ is as follows:

5 $$\begin{aligned} p_r = R(s, a, s') + \varepsilon \end{aligned}$$

where, R(s, a, s’) represents the immediate reward, and $\epsilon$ is a small positive constant that ensures all experiences have non-zero probabilities of being selected.

During different stages of training, the influence of $p_R$ and $p_t$ on the current network constantly changes. However, specifying fixed or linear priority weights artificially would ignore interaction between agent and environment. To accurately evaluate the value of experiences at different stages, we propose a mechanism for updating dynamically priority weights. This method adjusts the weights based on the importance of each criterion to the experience pool.

We sample a batch of size *M* from the experience pool *t* times and the total number of experiences available is *N*. To determine the priority of each sampled experience, we first calculate $p_r$ and $p_t$ based on the current network. Then, we get the actual score $\mu _j$ for each recent priority, which represents the average priority level of the experience pool. The calculation of $\mu _j$ is as follows:

6 $$\begin{aligned} \mu _j = \frac{{\sum _{i=1}^{T} \delta _{ij}}}{{N}} \end{aligned}$$

where, $\delta _{ij}$ is the priority of the i-th experience under the j-th criterion. For the newly sampled batch, the importance $S_j$ for each priority criterion is calculated based on the average priority level of the experience pool $\mu _j$ with the following formula:

7 $$\begin{aligned} S_j=\ P\ (\ \delta _{ij}\ >=\ \mu _j)\ \end{aligned}$$

where, $S_j$ is the estimation of the advantage probability of each priority criterion relative to average priority level. If the probability of a criterion in the batch is higher than the feedback information $\mu _j$, it indicates that the criterion is currently more important compared to others.

Based on $S_j$, we further calculate the influence factor $F_j$ for each priority criterion as follows:

8 $$\begin{aligned} F_j = e^{dS_j} - 1 \end{aligned}$$

We calculate the weight $W_j$ for each criterion according to $F_j$. The calculation formula is as follows:

9 $$\begin{aligned} W_j = \frac{{F_j}}{{\sum _{k=1}^{N} F_k}} \end{aligned}$$

By using this formula, we can get the weight $W_j$ for each criterion, which reflects the relative importance of each criterion in the learning process. This allows the agent to prioritize and focus on more meaningful experience, enabling effective learning.

After the new priority weights have been calculated, the experience is re-prioritized and saved to the experience pool. The new priority calculation formula is as follows:

10 $$\begin{aligned} P = \sum _{j} p_j \cdot W_j \end{aligned}$$

In summary, we propose an experience replay algorithm that combines TD-error and immediate reward with dynamically updated weights. When a batch is sampled from the experience pool, the agent updates the network parameters and evaluates the relative importance of each priority criterion in the context of the current network and the average level of the experience pool. After calculating the priority weights, the experience priority is updated. The detailed algorithm is shown in Algorithm 1.

### Ethics approval

This article does not involve any studies conducted by the authors with human participants or animals.

**Algorithm 1:**
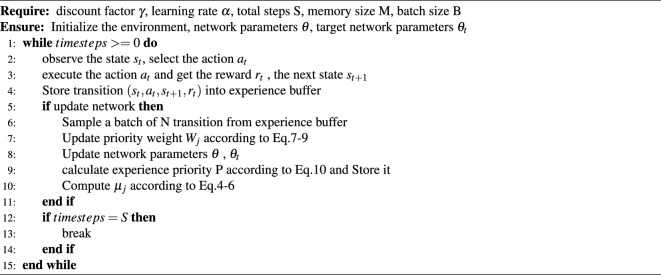
*PERDP*

**Table 1:** Comparison of time steps required for model convergence.

Condition	PERDP	PER	RT
MsPacman	55.75k	69.96k	73.36k
Alien	42.24k	59.63k	70.55k
UpNDone	16.87k	27.27k	46.18k

**Figure 3:**
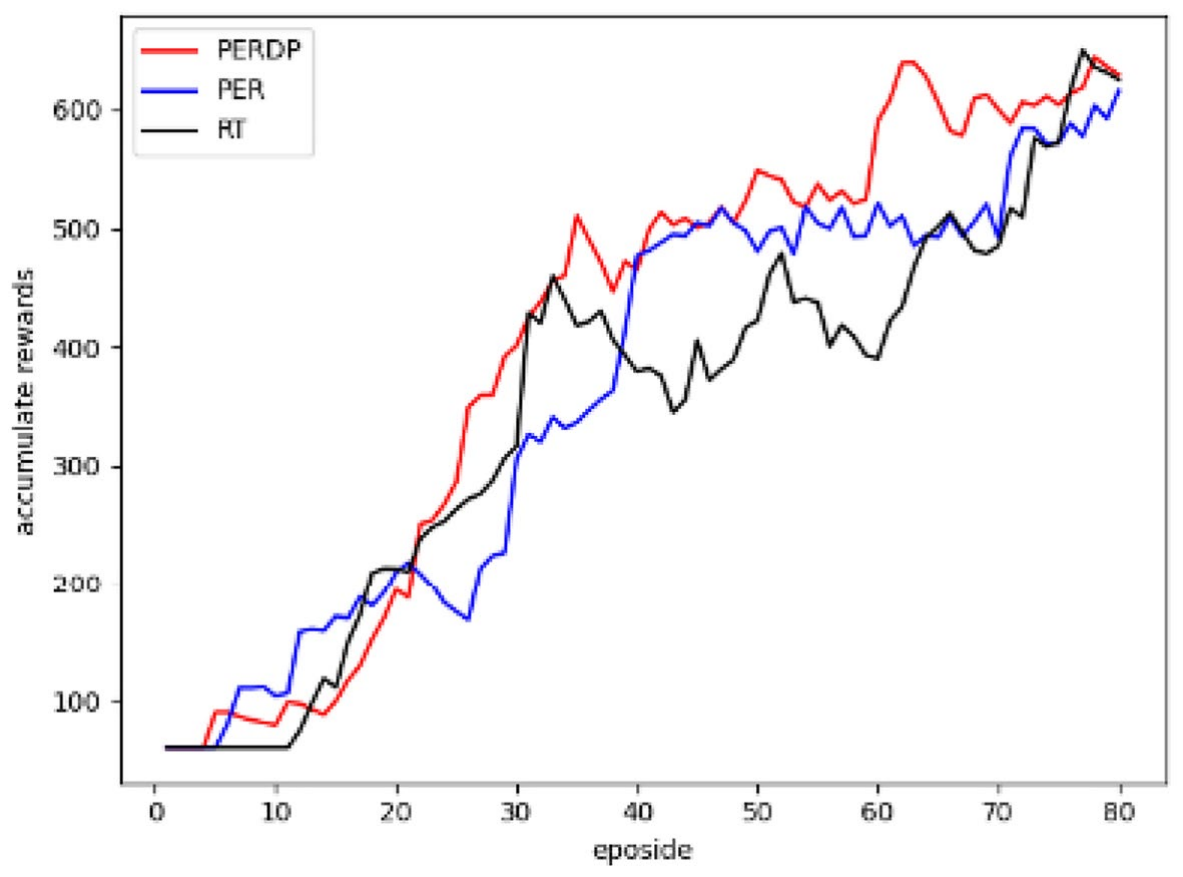
Evaluation results of the MsPacman environment.

**Figure 4:**
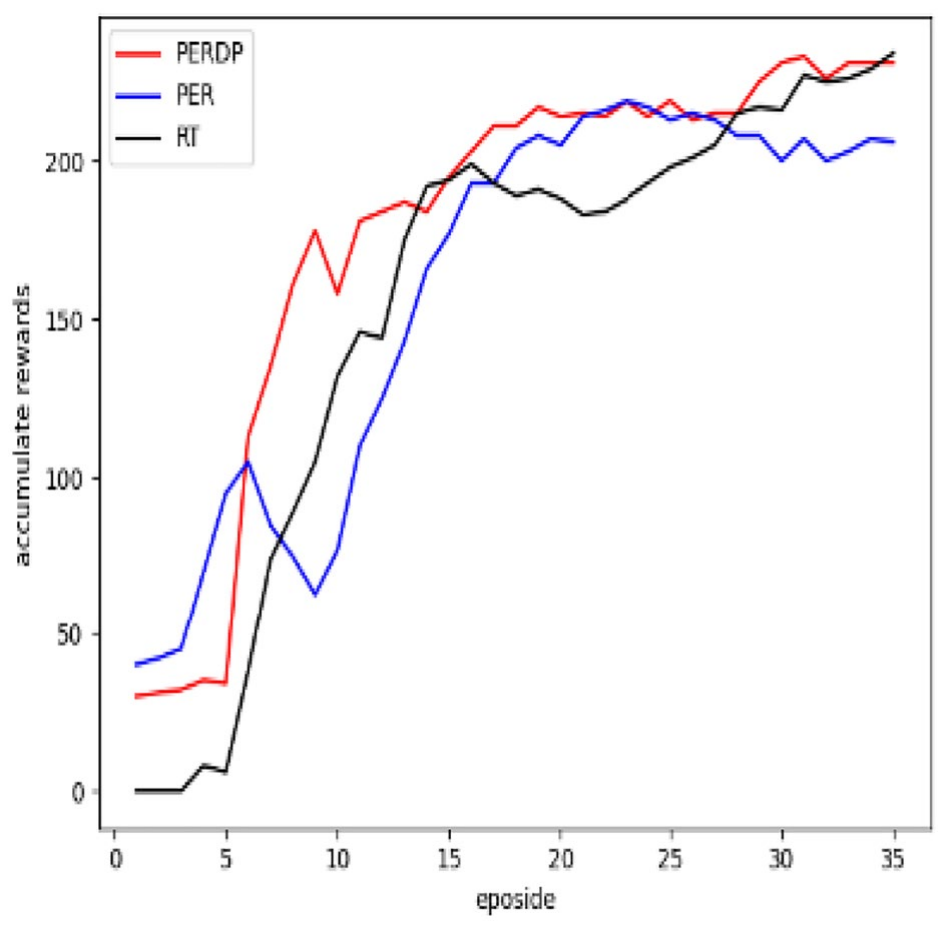
Evaluation results of the Alien environment.

**Figure 5:**
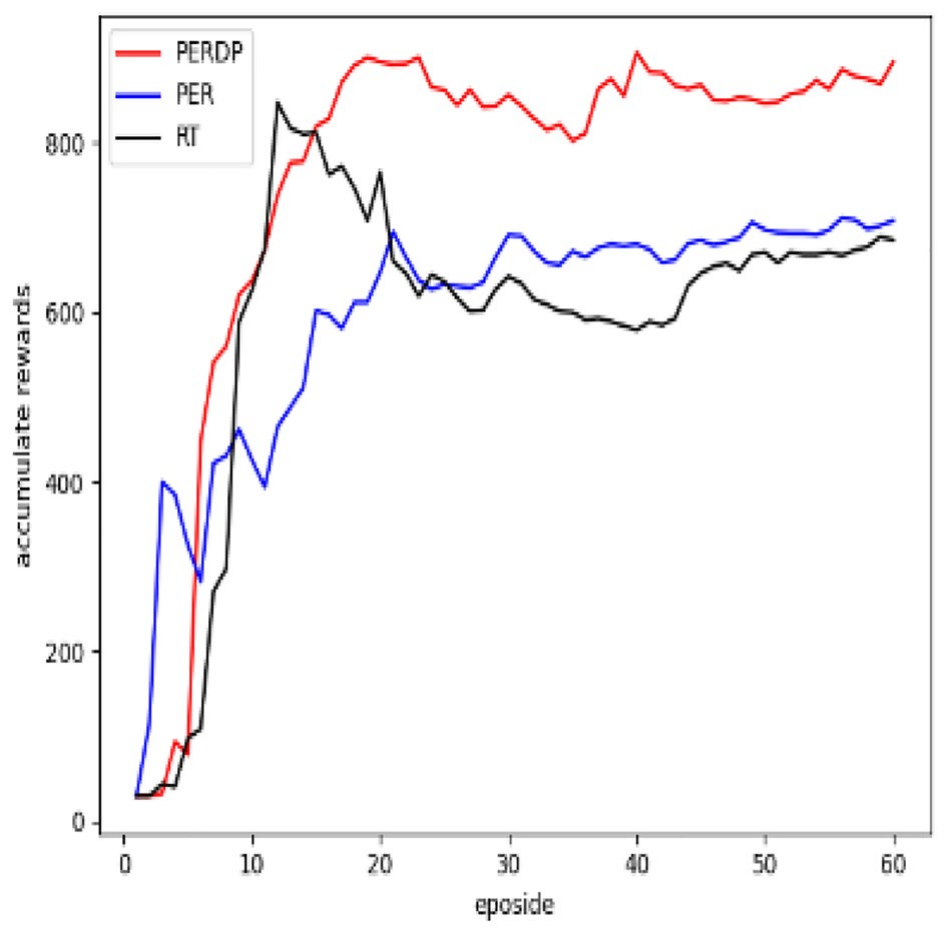
Evaluation results of the UpnDown environment.

## Experience

We apply the proposed algorithm, PERDP , to the SAC model and conduct experiments in video games environment. The objective is to validate that PERDP can converge rapidly and achieve optimal performance with fewer training steps. For comparison, we select two improved algorithms for DQN as baselines: PER^5^ and RT (Reward and td error parameter) experience replay^13^. In total, three algorithms are appear in the experiment, as follows:

1. PER-prop: This algorithm prioritizes experiences based on TD-error, which is a modified version of the DQN algorithm with uniform sampling. The TD-error serves as an indicator of the importance of an experience.
2. RT: This method is a prioritized experience replay algorithm that combines TD-error and reward. The weight between these priorities is fixed.
3. PERDP: This approach is an improved algorithm of RT, which adjusts dynamically the weights .

### Experience detail

To ensure the fairness of the experiments, we conducted all algorithms in the same environment with identical hyperparameters. Our algorithm is implemented on the basis of SAC-Discrete using PyTorch. The following hyperparameters were used:learning rate is 0.0003, gamma is 0.99, hidden layer of policy network is a fully connected neural network with 512 neurons, number of input neurons is state space, number of output neurons is action space.In contrast, the batch size was set to 32, the experience pool capacity to 50,000, and the target network update interval to 1000 time steps. Additionally, each algorithm use a soft update strategy. SAC-Discrete using PyTorch code is vailable from https://github.com/ku2482/sac-discrete.pytorch.

### Atari experience

#### Experimental results

In the evaluation of the PERDP algorithm, we chose three Atari environments to test its performance. These environments were carefully selected to provide a diverse range of challenges for our algorithm (More experimental results can be found in the Supplementary Figs. S1–S4). The cumulative reward evaluation results of the three algorithms were presented in Figs. 3, 4 and 5, which clearly demonstrated the superior performance of the PERDP algorithm. However, it is worth noting that our algorithm did not immediately outperform the other two methods. In fact, at the very beginning of the training process, its performance was similar to that of the other two algorithms. This can be attributed to the fact that the agent received sparse rewards with a lot of noise, making it difficult for the PERDP algorithm to accurately measure the overall value of the experience pool. However, PERDP has a more rapid boost at the early stage of training process, proving its capacity to more precisely evaluate the information contained in experiences, allowing agent to quickly learn the optimal policy. This is a key advantage of the PERDP algorithm over the other two methods, which showed the same trend but lacked the same level of performance in the later stages of training.To further support our findings, we also included Table 1, which highlights the good performance of PERDP with fewer steps.Furthermore, we found that our proposed algorithm tends to has better performance in later stages. This is because the agent can capture more suitable priority criterion in complex environments, leading to optimal decision making.

As for PER algorithm, it can be found from the experimental results that it initially outperformed the other two algorithms, but its learning speed gradually slowed down, and in the end, it performed worse than the PERDP algorithm. In the very beginning of training, the PER algorithm excels at obtaining rewards quickly. By prioritizing experiences with high TD-errors, the algorithm can quickly sample advantageous experiences and update its policy accordingly. However, as the training progresses and the policy gradually approaches the optimal network, the learning speed of the PER algorithm tends to slow down. This is because the PER algorithm tends to focus on experiences with high TD-errors, while ignoring other samples that may have potential value. As a result, the model may overfit to these experiences, potentially leading to it performing poorly in later stages and even getting stuck in local optima. On the other hand, the RT algorithm, which shares similarities with PERDP, also exhibits fast learning capabilities. However, RT algorithm performs even worse in the later stages of training. RT algorithm fails to notice the changing trend in the importance of priority criteria. It just assigns a fixed weight to each priority criterion regardless of its relevance to the environment, without adjusting strategy according to environmental changes. So RT algorithm likely leads to the algorithm struggling to adapt to new requirements in dynamic environments, resulting in lower performance. This limitation can hinder the algorithm’s ability to learn and perform optimally in dynamic environments.

#### Result analysis

**Figure 6:**
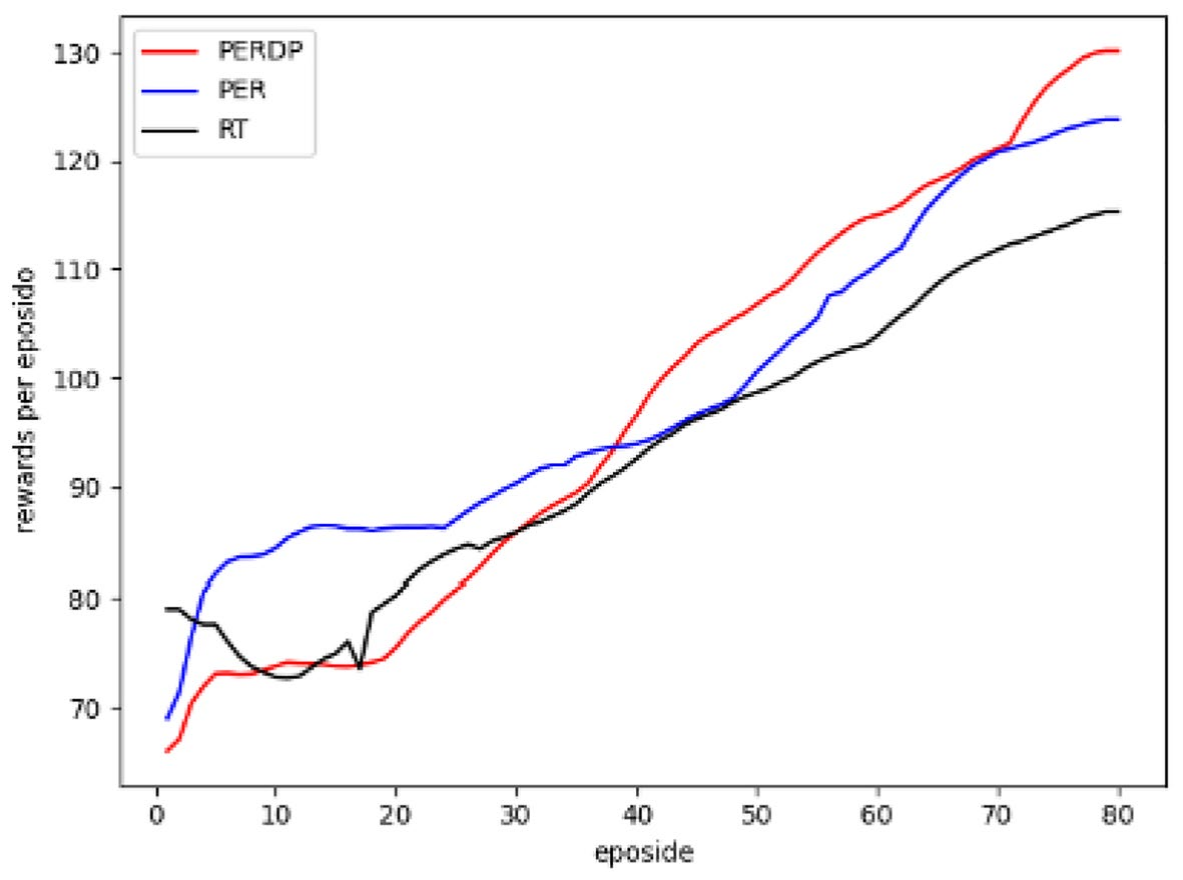
This is a comparison of the average rewards among three methods.

In order to better understand the PERDP algorithm, we analyzed the average reward and state entropy. Figure 6 illustrates the average reward obtained by the agent. It can be seen that the PER achieves higher average rewards in the early stages due to its focus on exploring the positional information of the environment. Despite the fact that the agent traversed many high-value trajectories, PER did not pay attention to these rewarding experiences and instead focused on TD-error. As training going on, TD error becomes less important for policy networks that have predictive ability, instead performing similar to uniform sampling. So they often perform poorly in the later stages. On the other hand, PERDP evaluates the priority of experiences from multiple perspectives in the later stages and train the most valuable experiences from the experience pool. This enables the model to maintain an advantage over the other two methods. In different environments, each standard priority tends to stabilize finally, similar to uniform sampling. However, PERDP can assess the contribution value of priority standards during the training process, enabling the agent to learn more.

**Figure 7:**
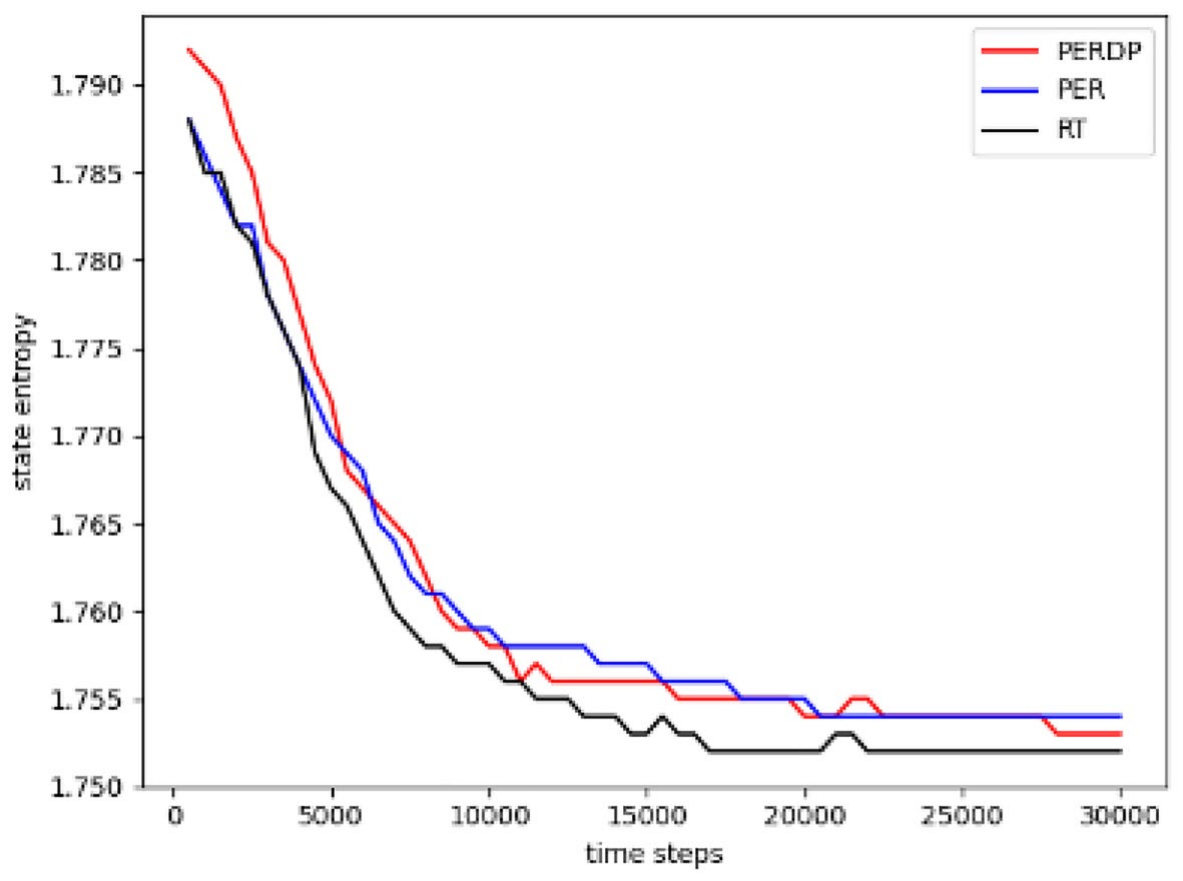
This is a comparison of the entropy among three methods.

The curve depicting the change of state entropy over the training steps is shown in Fig. 7. It can be observed that the state entropy of PERDP is higher than the other two methods in the initial stages, while PER and RT exhibit similar trends. This indicates that our approach ensures that the agent encourages exploration. Subsequently, the state entropies of the methods gradually decrease and approach each other as the networks begin to have some predictive ability.

## Discussion and conclusion

In this study, we introduce a novel method , PERDP, to improve sampling efficiency. PERDP combines TD-error and immediate reward to select more important experiences from the experience pool. Besides, we introduce a dynamic priority adjustment framework. When calculating the total priority of experience, weight of priority are calculated based on the current experience pool by analyzing the importance of both priority criterion. Then we calculate the overall priority of the experience based on the weight. This approach prevents the issue of the agent inaccurately estimating the value of experience in dynamic or complex environments. So the agent can learn more from more valuable experiences. The experimental results obtained from discrete action control tasks demonstrate that PERDP has faster convergence speed and better performance. In the future, we plan to explore other efficient priority criteria and integrate it into the framework to accurately assess the value of experience. Meanwhile, we found that PERDP still faces challenges in sparse reward environments. So, we would like to extend our work to solve sparse reward tasks.

### Supplementary Information

Supplementary Figures.

## Data Availability

The datasets used and/or analyzed during the current study are available from the corresponding author on reasonable request.
