# Supplementary material for: Prioritized experience replay based on dynamics priority
Source: Sci Rep. 2024 Mar 12;14:6014. doi: 10.1038/s41598-024-56673-3 (PMC10933423; doi:10.1038/s41598-024-56673-3)
Supplement: Supplementary file 1 — Supplementary Figures. [file 41598_2024_56673_MOESM1_ESM.pdf]

1 Supplementary

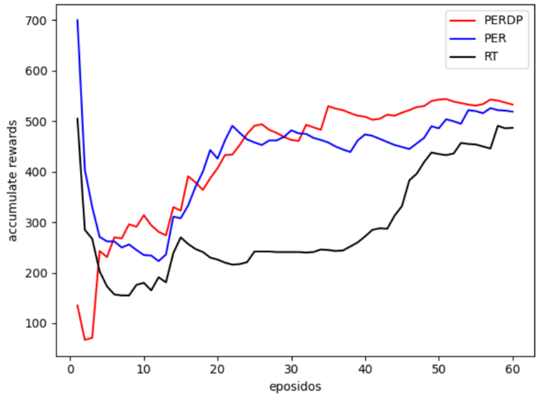

Supplementary Fig S1: AirRaid environment

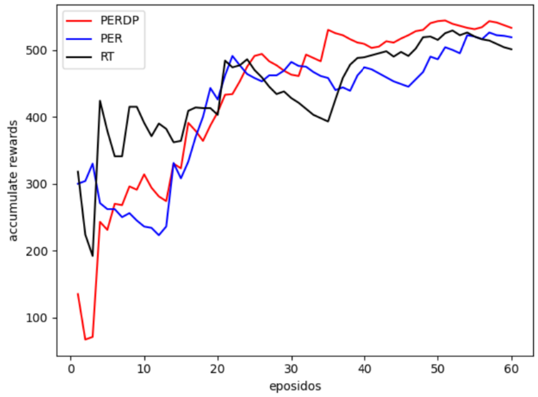

Supplementary Fig S2: Asterix environment

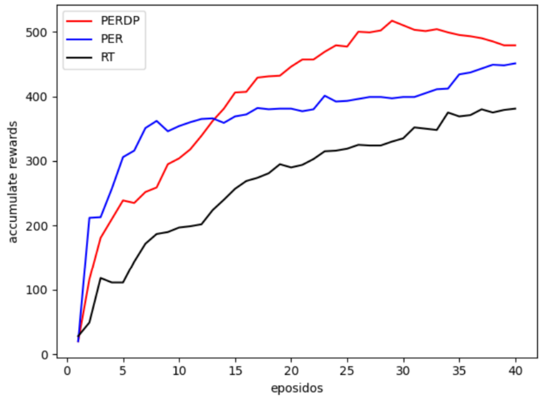

Supplementary Fig S3: Phoenix environment

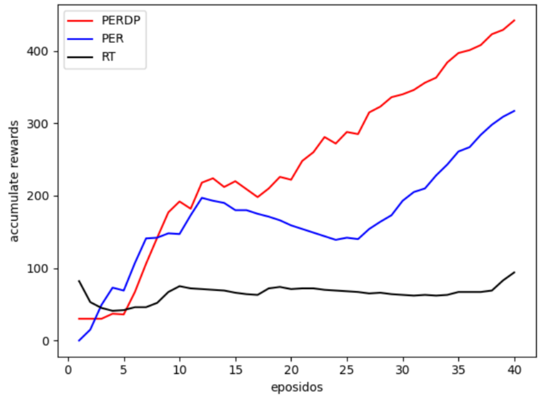

Supplementary Fig S4: Pooyan environment
